# Correlation between Family Environment and Suicidal Ideation in University Students in China

**DOI:** 10.3390/ijerph120201412

**Published:** 2015-01-27

**Authors:** Hui Zhai, Bing Bai, Lu Chen, Dong Han, Lin Wang, Zhengxue Qiao, Xiaohui Qiu, Xiuxian Yang, Yanjie Yang

**Affiliations:** 1 Department of Medical Psychology, Public Health Institute of Harbin Medical University, Harbin 150081, China; E-Mails: zhaihui323@sina.com (H.Z.); handong88222@126.com (D.H.); wanglin19860451@163.com (L.W.); qiaozhengxue0@163.com (Z.Q.); qiuxiaohui@foxmail.com (X.Q.); xiuxian0619@163.com (X.Y.); 2 Department of Cardiology, First Affiliated Hospital of Harbin Medical University, Harbin 150081, China; E-Mail: baibing2910@163.com; 3 Department of Endocrinology, Peking Union Medical College Hospital, Beijing 100730, China; E-Mail: lululululucy@163.com

**Keywords:** China, university students, family environment, suicidal ideation

## Abstract

*Background*: This study investigated the association between suicidal ideation and family environment. The sample included 5183 Chinese university students. A number of studies on suicidal ideation have focused on individuals rather than families. This paper reviews the general principles of suicidal ideation and the consequences resulting from the family environment. *Methods*: This study used six different colleges as the dataset, which included 2645 males and 2538 females. Students were questioned with respect to social demographics and suicidal ideation factors. The data were analyzed with factor and logistic analyses to determine the association between suicidal ideation and poor family environment. *Results*: The prevalence of suicidal ideation was 9.2% (476/5183). Most participants with suicidal ideation had significant similarities: they had poor family structures and relationships, their parents had unstable work, and their parents used improper parenting styles. Female students were more likely to have suicidal thoughts than male students. *Conclusions*: This study shows that suicidal ideation is a public health issue among Chinese university students and demonstrates the importance of considering the family environment when examining university students’ suicidal ideation. Understanding family-related suicidal ideation risk factors can help to predict and prevent suicides among university students.

## 1. Background

Suicide has unique epidemiological characteristics that make it an important public concern. A recent national estimate reported an annual suicide rate of 23 per 100,000 people in China [1]. Suicide thus accounts for 3.6% of all deaths in China and ranks as the fifth most common cause of death. Suicide among university students accounts for 19% of all deaths and is the leading cause of death among that population [1]. Suicide among university students has become a serious public health problem, and the rise in the student suicide rate has led to an increasing number of studies on the factors that explain suicidal behavior [2,3,4,5,6]. Suicidal ideation among students is defined as the wish, thought or desire to take one’s own life violently due to a variety of internal and external causes, such as personality, undesirable emotions and school life [7]. Information on suicidal ideation can be utilized to predict suicide and can act as a guide for suicide prevention [8]. This paper will focus on suicidal ideation as a key element to determine the factors related to suicide.

Previous studies on university students’ suicidal ideation have been overly concerned with school environments, students’ undesirable behavior and psychological symptoms [9,10,11]. Few studies have investigated how family environments act upon university students’ suicidal ideation, and the research approaches have been rather limited. A number of studies have shown that the family environment significantly affects suicidal behavior among university students [12,13]. The family is a social-life community of relatives established by marital, blood, and adoptive relationships; it is the most intimate group and has a profound effect on its members. Family characteristics vary across cultures and nations. China has a unique marriage system, employment structure and parenting style, and these specific characteristics may affect family members. 

When studying family risk factors for suicidal ideation among university students in China, parental structure and relationships may be important predictors. Traditional Chinese values reject divorce and view it as a shameful event. Therefore, parents prefer to try to live together with difficulty rather than to divorce. This preference can give rise to poor parental relationships. Over the last three decades, China has experienced steadily increasing divorce rates, from 0.4 per 1,000 persons in 1985 to 1.85 per 1000 in 2009 [14]. Poorer parental relationships and higher divorce rates cause misfortune and psychological distress for students, which may cause students to experience suicidal ideation [15,16].

Parents’ employment structure, such as work instability, may also be considered an important variable when assessing suicidal ideation in university students. Currently, China’s society is in a transition period. To adapt to the change of the market economy, Chinese enterprises have downsized a large portion of existing employees and have adjusted the contract system to include new employees. This approach has resulted in a loss of work for some Chinese people. The parental salary is the only income of a family, and an unstable salary can affect the family’s economic status. Many foreign studies of university students have indicated that low family income leads to thoughts of suicide [17]. 

Parenting style is also strongly associated with students’ suicidal ideation. In Chinese families, there is a more rigid hierarchical structure than in families in other countries [18]. University students are more apt to obey their parents due to elders’ parentalism. Parents force students to follow their demands, and they do not respect students’ thoughts and desires. In parents’ minds, the only parenting behavior that is good for students is to satisfy their need for clothing, food, housing and transportation. If students act against their parents’ will, they are punished or even scolded and beaten. Chinese parents often have highly ambitious goals for their children, which are often manifested in parental discipline of their children’s studying. Parents burden their students with the pressure to learn. Studies in other countries have shown that suicidal ideation is associated with parenting methods [19]. It may be that improper parenting increases the risk of suicidal ideation in children.

Given the unique family environment of Chinese university students, a better understanding of the relationship between the family environment and suicidal ideation is needed to provide and implement targeted interventions to prevent suicidal ideation among university students. Therefore, in this study, we attempted: (1) to determine the prevalence of suicidal ideation among university students, (2) to determine whether the family relationship, parenting style and stability of parents’ work were associated with students’ suicidal ideation, and (3) to identify the predictors of family factors of suicidal ideation and adopt targeted interventional measures to address the suicidal ideation of university students.

## 2. Methods

### 2.1. Study Population

We collected data from Harbin, the capital of Heilongjiang Province in China. Harbin is the center of the northeastern area in terms of politics, economics and culture. It has a total area of approximately 53,840 square kilometers, and the total registered population is 994 million. In China, two types of universities exist: national key universities and ordinary universities. There are three national key universities and 11 ordinary universities in Harbin. National key universities are state funded, and competition among their students is intense. Ordinary universities are provincially funded, and their students experience less competition. Students in these 14 universities come from all over the country and constitute a population of approximately 274,041.

### 2.2. Sample Size and Sampling Technique

To obtain a representation of universities in China, we first selected two national key universities (Harbin Institute of Technology and Harbin Engineering University) and four ordinary universities (Harbin Medical University, Harbin Normal University, Heilongjiang University and Heilongjiang Institute of Technology) via a stratified random clustering of all 14 universities in Harbin. Then, we estimated the sample size based on the number of students in each university and chose 6000 students. Stratified sampling was used to select full-time students. The samples were stratified into first-year, second-year, third-year, fourth-year and postgraduate fifth-year students. We randomly selected classes from each grade, and all students in the selected classes were invited to participate in the survey.

An exploratory design was employed to gather quantitative data from the universities. Participants who were identified as experiencing suicidal ideation were categorized into a suicidal ideator group (476 suicidal ideator students; 287 female, 189 male). These individuals were invited to complete the social demographic and family-associated factor questionnaires and the Adolescent Self-Rating Life Events Checklist (ASLEC). None of the participants suffered from any type of personality/psychiatric disorder or organic brain lesions.

### 2.3. Procedure

Students were recruited personally through the university. The surveys were administered in each participant’s classroom after class. Before the data collection, the schools and the participants gave consent to complete the questionnaires. All participants gave written informed consent after the nature of the study was explained to them. The study was approved by the Ethics Committee of the Department of Psychology at Harbin Medical University. To help the participants understand and complete the questionnaires within 15 min, the research assistants presented a short introduction to address the general aims and confidentiality of the research. The research assistants explained the entire administrative process and answered queries raised by the participants. All questionnaires were collected upon completion.

### 2.4. Assessment

Basic socio-demographic information and family-related factors were collected via the social demographic questionnaire. In the questionnaire, we asked the participants to indicate their gender, their age, their university type, the number of days they were affected by insomnia, whether they smoked or drank, whether they were affected by disease, their psychological status and their family status. The self-report survey on the family identified the participants’ economic status, their annual income, their parents’ current situation, their parents’ relationship, their relationship with their parents, their parents’ education level, their parents’ work and whether they were the only child.

The Adolescent Self-Rating Life Events Checklist (ASLEC) was used in the survey. The ASLEC contains 27 items that assess whether certain life events have happened and the respondent’s perception of the severity of these life events (none, mild, moderate, severe, extremely severe). We used 24 and 25 items to assess whether students were often scolded or beaten by their parents or received pressure from their parents related to learning.

Suicidal ideation was tested by a two-tier process. The first-tier screen used the suicide item in the Patient Health Questionnaire depression scale (PHQ-9). This item assesses whether participants have thought in the previous 2 weeks that they would be better off dead. Students who reported suicidal ideation for a few days or longer were selected for the mini interview. The students were asked, “Have you ever thought about killing yourself?” Serious suicidal ideation, suicidal plans and suicide attempts were also assessed in line with the National Comorbidity Survey [20]: “Have you ever seriously thought about killing yourself?” “Have you ever made a plan for killing yourself or even taken measures to do something for this plan?” All questions showed significant reliability. Students who agreed with any question in this section were identified as having suicidal ideation.

### 2.5. Data Analysis

We used SPSS18.0 to analyze the data. Descriptive statistics were used to determine the quantity and percentage distributions. To obtain the association between dysfunctional family dynamics and suicidal ideation, we first used a factor analysis and then included the significant factors in a multivariate logistic regression. Throughout the process, the *p* value was set at 0.05, and 95% confidence intervals were calculated.

## 3. Results

### 3.1. Participants’ Socio-Demographic and Household Data

A total of 6000 questionnaires were distributed, and the response rate was 91.3%. Ultimately, we collected 5183 valid questionnaires after excluding invalid questionnaires. There were more males (51.04%) than females (48.96%) in the sample, which reflects the gender distribution among the general population. The average age of the participants was 21.20 years (SD = 2.03) and ranged from 16 to 43 years. The study sample was composed of 2645 men and 2538 women. Of this sample, 476 participants reported suicidal ideation, and 4707 participants reported not having suicidal ideation. Furthermore, 1558 students were from national key universities, and 3625 students were from ordinary universities; however, university type did not predict suicidal ideation among these Chinese university students. The characteristics of the subjects are shown in Table 1. Based on self-reports, 1427 students had insomnia, 482 students were smokers, 1712 students were alcohol abusers, 682 were affected by disease, and 914 had psychological problems. Sex, age, insomnia, drinking, disease and psychological problems were all associated with suicidal ideation. No family factors except being an only child were correlated with suicidal ideation.

**Table 1:** Distribution and family controlled variables of the Chinese university student sample according to suicidal ideation.

Control variables	Total100 (5183)	Suicidal Ideator	Non-Suicidal Ideator	χ^2^/t	*p*
Sex				26.91	<0.0001
Female	48.97% (2538)	60.29% (287)	47.82% (2251)		
Male	51.03% (2645)	39.71% (189)	52.18% (2456)		
Age	21.32 ± 2.20	21.07 ± 1.83	21.34 ± 2.23	3.07	0.0023
University type				0.91	0.34
Key element	30.06% (1558)	28.15% (134)	30.25% (1424)		
Ordinary	69.94% (3625)	71.85% (342)	69.75% (3281)		
Insomnia				43.07	<0.0001
No	72.47% (3756)	59.66% (284)	73.76% (3472)		
Yes	27.53% (1427)	40.34% (192)	26.24% (1235)		
Smoke				0.002	0.96
No	90.70% (4701)	90.76% (432)	90.69% (4269)		
Yes	9.30% (482)	9.24% (44)	9.31% (438)		
Drink				8.66	0.003
No	66.97% (3471)	60.92% (290)	67.58% (3181)		
Yes	33.03% (1712)	39.08% (186)	32.42% (1526)		
Disease affected				39.85	<0.0001
No	86.84% (4501)	77.52% (369)	87.78% (4132)		
Yes	13.16% (682)	22.48% (107)	12.22% (575)		
Psychologically affected				132.06	<0.0001
No	82.37% (4269)	63.24% (301)	84.30% (3968)		
Yes	17.63% (914)	36.76% (175)	15.70% (739)		
Only-child				10.63	0.001
No	28.03% (1453)	21.64% (103)	28.69% (1350)		
Yes	71.97% (3730)	78.36% (373)	71.32% (3357)		
Economic status				2.16	0.339
Good	7.6% (394)	5.5% (26)	6.9% (323)		
Mediate	67.6% (3503)	66.8% (318)	67.7% (3185)		
Poor	25.7% (1331)	27.7% (132)	25.5% (1199)		
Annual economic income				4.48	0.107
≤10000	32.5% (1684)	5.5% (26)	6.9% (323)		
≤20000	49.4% (2559)	66.8% (318)	67.7% (3185)		
>20000	18.1% (940)	27.7% (132)	25.5% (1199)		
Father’s educational level				2.48	0.479
Primary and lower	12.6% (653)	13.7% (65)	12.5% (588)		
Middle school	27.0% (1398)	27.3% (130)	26.9% (1268)		
High school	56.4% (1924)	34.0% (162)	37.4% (1762)		
College and above	23.3% (1208)	25.0% (119)	23.2% (1089)		
Mother’s educational level				1.42	0.700
Primary and lower	20.3% (1052)	21.2% (101)	20.2% (951)		
Middle school	27.1% (1406)	28.6% (136)	27.0% (1270)		
High school	34.7% (1796)	32.4% (154)	34.9% (1642)		
College and above	17.9% (929)	17.9% (85)	17.9% (844)		

### 3.2. Family Factors Extracted from Factor Analysis

Three factors with eigenvalues >1.0 were identified and accounted for 60.64% of the overall variance. These factors explained 21.79%, 19.68% and 19.17% of the total variance. The factor loadings are shown in Table 2. Factor 1 was “poor family structure and relationships”, including poor parental relationships and parental divorce. The second factor referred to “parents’ unstable work”, including that of both fathers and mothers. The third factor (improper parenting style) included being scolded and beaten by parents, receiving learning pressure from parents and having poor relationships with parents.

**Table 2:** Loadings on first rotated principal components for all university students.

Family factors	Factor1	Factor2	Factor3
Poor parental relationship	0.78 **^a^**	−0.33	−0.08
Parental divorce	0.76 **^a^**	−0.36	−0.12
Unstable paternal work	0.29	0.71 **^a^**	−0.32
Unstable maternal work	0.33	0.68 **^a^**	−0.35
Scolded and beaten by parents	0.17	0.29	0.68 **^a^**
Learning pressure from parents	0.19	0.29	0.71 **^a^**
Poor relationship with parents	0.29	−0.00	0.36 **^a^**

**^a^** Indicate the largest loadings on each component.

### 3.3. Family Factors Associated with Suicidal Ideation among Chinese University Students: Chi-Square Analysis 

As shown in Table 3, in a comparison of suicidal ideators and non-suicidal ideators, family-related variables were found to be common among all suicidal ideators. 

**Table 3:** Family associated factors of Chinese university students, with and without suicidal ideations.

Family Factors	Total100 (5183)	Suicidal Ideator	Non-Suicidal Ideator	$\chi^{2}$	*p*
Scolded and beaten by parents				23.96	<0.0001
No	84.08% (4358)	76.26% (363)	84.87% (3995)		
Yes	15.92% (825)	23.74% (113)	15.13% (712)		
Learning pressure from parents				50.01	<0.0001
No	65.52% (3396)	50.84% (242)	67.01% (3154)		
Yes	34.48% (1787)	49.16% (234)	32.99% (1553)		
Poor parental relationship				19.71	<0.0001
No	95.58% (4954)	91.60% (436)	95.98% (4518)		
Yes	4.42% (229)	8.40% (40)	4.02% (189)		
Poor relationship with parents				53.47	<0.001
No	89.74% (4651)	80.04% (381)	90.72% (4270)		
Yes	10.26% (532)	19.96% (95)	9.28% (437)		
Parental divorce				6.47	0.01
No	91.36% (4735)	88.24% (420)	91.67% (4315)		
Yes	8.64% (448)	11.76% (56)	8.33% (392)		
Unstable paternal work				5.61	0.0178
No	95.95% (4973)	93.91% (447)	96.15% (4526)		
Yes	4.05% (210)	6.09% (29)	3.85% (181)		
Unstable maternal work				8.97	0.0027
No	96.20% (4986)	93.70% (446)	96.45% (4540)		
Yes	3.80% (197)	6.30% (30)	3.55 (167)		

### 3.4. Family Factors Associated with Suicidal Ideation among University Students in China: Logistic Regression Analysis

Table 4 presents the logistic regression analysis results for suicidal ideation among university students in China. Group-specific odds ratios are shown in the second column of Table 4. In the third column, three factors were evaluated for their association with suicidal thoughts when demographic/family factors variables were controlled. In the fourth column, the three factors were not only adjusted for demographic/family factor variables but also adjusted for each other. After all confounders and three family factors were added to the model, suicidal ideation was still associated with these three factors.

**Table 4:** Suicidal ideation and correlated family factors using univariate and multiple logistic regression analysis.

Family Factors	Unadjusted Odds Ratios with 95% CI	Adjusted Odds Ratios with 95% CI	
		Demographic Model ^a^	Family Factors Model ^b^
Factor1	1.179 (1.092, 1.273) **^c^**	1.124 (1.038, 1.217) **^d^**	1.124 (1.038, 1.217) **^d^**
Factor2	1.158 (1.072, 1.250) **^c^**	1.096 (1.010, 1.188) **^e^**	1.101 (1.015, 1.195) **^e^**
Factor3	1.455 (1.338, 1.582) **^c^**	1.399 (1.281, 1.528) **^c^**	1.402 (1.284, 1.532) **^c^**

Notes: ^a^ The odds ratios for risk factors were adjusted only for demographic/family factors variables; ^b^ The odds ratios for risk factors were all adjusted for each other in addition to demographic/family factors variables; ^c^ *p* < 0.001; ^d^ *p* < 0.01; ^e^ *p* < 0.05.

## 4. Discussion

The findings in this study suggest that Chinese university students, particularly females, have a higher risk of suicidal ideation [21]. More importantly, our results show that poor family relationships, parents’ unstable work and improper parenting styles have a strong correlation with suicidal ideation among university students. The implications of these analyses are self-evident and far-reaching. Considering the family factors, more effort should be applied to prevent suicidal ideation. This study provides evidence of the importance of the family relationship, parents’ work and parenting methods in reducing the risk of suicidal ideation among university students.

### 4.1. Prevalence of Suicidal Ideation and Gender Difference

The survey reported that 9.2% of students had suicidal ideation. This result is relatively lower than the prevalence of suicidal ideation in many other countries, such as the United States, where it is estimated to be 12% [5]. The rates of suicidal ideation are also higher in other Asian studies than the rate found in the present study. In India, the lifetime prevalence of suicidal ideation is 11.7%, and in Pakistan, the overall rate of suicidal ideation is 31.4% [22,23]. Thus, the prevalence of suicidal ideation among university students seems to vary across regions. Our findings are consistent with the conclusions of previous studies that found that females are more prone to suicidal ideation than males are. However, our findings are inconsistent with studies that found no gender differences in suicidal ideation among university students [24,25]. 

### 4.2. Poor Family Elements as Risk Factors for College Students’ Suicidal Ideation

#### 4.2.1. Poor Family Structure and Parental Relationship

Consistent with other studies, this study found strong associations among parental divorce, a poor relationship between parents, and students’ suicidal ideation. For many students, having divorced parents or parents with negative relationships disrupts the cohesiveness of the family, which can result in a low level of well-being. According to Sussie’s study, students who had close family relationships reported less suicidal ideation [26]. 

A longitudinal panel study of students 12 to 24 years of age showed that students from divorced families had more suicidal ideation [27]. Previous studies have shown that parental divorce leads to anxiety and mood disorders among students [28,29], and suicidal ideation is commonly seen in anxiety disorders and mood disorders, including depression [30]. A negative physical and psychological state may be one of the reasons for university students’ suicidal ideation. 

#### 4.2.2. Unstable Parental Work

In our study, suicidal ideation increased when the participants’ parents had unstable work. This finding is inconsistent with a survey that found that youth can gain happiness from parental unemployment because unemployed parents may have more time to spend with their children, thus increasing children’s well-being [31]. However, that study assessed boys and girls aged 11–15 years, whereas university students are independent adults. Fluctuating family economic status may be one of the most important factors that determines quality of life and affects students’ mental health, and parents’ unstable work may lead to a poor family economic status. Andrews and Wilding [17] reported that financial difficulties can increase college students’ level of anxiety and depression, which may lead to increased suicidal behavior**.** McLoyd, Jayaratne, Ceballo, and Borquez [32] concluded that poverty negatively affects parenting and promotes poor socio-emotional adjustment among poor students, which adds to their psychological burden. Many foreign studies have established family economic status as a risk factor for suicidal ideation. Therefore, we suggest that appropriate interventions of help for unemployed parents may be useful in decreasing the occurrence of university students’ suicidal ideation.

#### 4.2.3. Improper Parenting Style

This study suggests that college students who have suicidal thoughts are more likely to have been subjected to improper parenting styles, including being scolded and physically punished by parents; to have felt learning pressure from parents; and to have had a poor relationship with their parents. Other surveys have also concluded that suicidal ideators are subjected to poor parenting methods [19]. 

A large number of previous studies have shown that college students who were victims of physical abuse were more likely to have suicidal thoughts. In Chinese culture, students’ greatest obligation is to their parents based on the deeply rooted belief that they are obligated to their parents for giving them life and caring for them. In addition, Chinese culture tends to attribute family violence to life’s misfortunes and encourages people to passively endure it [33]. These studies emphasize the deleterious consequences of parental abuse. Intense learning pressure is a stressful life event for college students. Both physical abuse and learning pressure are due to parent-child conflict, which is one of the most influential factors in suicidal ideation [34,35]. A study of 75 college students who experienced suicidal ideation reported that parent-student conflict was independently associated with suicidal ideation [36]. These findings suggest that comprehensive measures should be taken to reduce abusive parenting, decrease parents’ learning pressure and improve the parent-student relationship to prevent university students’ suicidal ideation. 

Although physical discipline by Chinese parents is prevalent and differs from discipline in foreign countries, the prevalence of Chinese university students’ suicidal ideation is relatively lower than that of students in other countries. The causes of Chinese students’ suicidal thoughts are complex. Culture plays an important role in explaining the suicidal ideation of Chinese university students who come from a Chinese family environment. All evidence suggests that a poor family environment is associated with the risk of suicidal ideation among Chinese university students. These students often view their family as passive and are dissatisfied with their family environment. In addition, female university students in China are more likely than males to have suicidal ideation.

## 5. Limitations

This study has a number of limitations. First, this study was a cross-sectional survey, which limits the causal relationship between family factors and suicidal ideation. Second, self-reported measures were used to collect data (e.g., suicidal ideation and family relationships) instead of scale tests in a more comprehensive approach, although we added a mini interview to the suicidal ideation test. Third, although we controlled for family factors, there may be unmeasured family variables among university students that contribute to suicidal ideation 

## 6. Conclusions

This study identified family aspects of university students’ suicidal ideation. Many factors that contribute to suicidal ideation have been identified. First, a poor family structure and parental relationship are positively correlated with suicidal ideation. Further research is needed to determine whether changes to poor family structure and parental relationships can decrease the presentation of suicidal ideation and how these factors can be prevented to help prevent suicide among students. Second, unstable parental work produces financial instability among university students and can lead to suicidal ideation. Third, university students who suffer from an improper parenting style may be at a high risk of suicidal ideation.

Suicide among university students accounts for 19% of all deaths and is the leading cause of death among that population [1]. Suicide is a significant problem for university students. Previous studies have overstated the role of the university in preventing suicide and suicidal ideation and have neglected the effect of family. Family is an important component of students’ environment. There is little doubt that family factors are closely related to students’ well-being and the prevention of suicidal ideation. The identification of a relationship between the family environment and suicidal ideation is a significant contribution to understanding the cause of suicide and developing interventions to enhance efforts toward suicide prevention. It is crucial to identify family risk factors. Regarding the key role of the family, our findings indicate that the family environment has a direct effect on suicidal ideation. This study also provides evidence that may contribute to measures to prevent suicidal ideation among students. We highlight the key role of the family in university students’ suicidal ideation.

With the complex transformations of the family environment, suicide prevention should be prioritized. Parents should do their best to provide their children with an enjoyable and intact environment to promote students’ health, growth and learning. Universities and society should encourage mutual respect, trust, and harmony among family members to maintain a harmonious family environment and to help parents find ways to prevent suicidal ideation among students. Finally, further scientific investigations are encouraged to test family interventions that could prevent students’ suicidal ideation.
